# Youth-onset Type 2 Diabetes: An Overview of Pathophysiology, Prognosis, Prevention and Management

**DOI:** 10.1007/s11892-024-01546-2

**Published:** 2024-07-03

**Authors:** Angela Titmuss, Sophy Korula, Brandy Wicklow, Kristen J. Nadeau

**Affiliations:** 1 Wellbeing and Preventable Chronic Diseases Division, Menzies School of Health Research, Charles Darwin University, Casuarina, PO Box 41096, Darwin, Northern Territory Australia; 2 Department of Paediatrics, Division of Women, Child and Youth, Royal Darwin Hospital, Darwin, Northern Territory Australia; 3 Paediatric Endocrinology and Metabolism Division, Paediatric Unit-1, Christian Medical College Hospital, Vellore, India; 4 Department of Paediatrics, Latrobe Regional Hospital, Traralgon, Victoria Australia; 5 Department of Paediatrics and Child Health, University of Manitoba, Winnipeg, Canada; 6 Children’s Hospital Research Institute of Manitoba, Winnipeg, Manitoba Canada; 7 Children’s Hospital Colorado, Aurora, Colorado USA; 8 School of Medicine, University of Colorado Anschutz Medical Campus, Aurora, Colorado USA

**Keywords:** Type 2 diabetes, Child, Youth, Insulin, Intergenerational, Hyperglycemia

## Abstract

**Purpose of review:**

This review explores the emerging evidence regarding pathogenesis, future trajectories, treatment options, and phenotypes of youth-onset type 2 diabetes (T2D).

**Recent findings:**

Youth-onset T2D is increasing in incidence and prevalence worldwide, disproportionately affecting First Nations communities, socioeconomically disadvantaged youth, and people of colour. Youth-onset T2D differs in pathogenesis to later-onset T2D and progresses more rapidly. It is associated with more complications, and these occur earlier. While there are limited licensed treatment options available, the available medications also appear to have a poorer response in youth with T2D. Multiple interacting factors likely contribute to this rising prevalence, as well as the increased severity of the condition, including structural inequities, increasing obesity and sedentary lifestyles, and intergenerational transmission from in-utero exposure to maternal hyperglycemia and obesity. Youth-onset T2D is also associated with stigma and poorer mental health, and these impact clinical management.

**Summary:**

There is an urgent need to develop effective interventions to prevent youth-onset T2D and enhance engagement of affected youth. It is also critical to better understand the differing phenotypes of youth-onset T2D, to effectively target treatments, and to address intergenerational transmission in high-risk populations.

## Introduction

The prevalence of youth-onset type 2 diabetes (YO-T2D), defined as T2D diagnosed before the age of 25 years, is increasing worldwide [1–3]. In 2021, an estimated 41,600 youth were newly diagnosed with T2D [4], with incidence varying widely around the world[3, 5]. Global incidence rate (per 100 000 population) increased from 117.2 in 1990 to 183.4 in 2019 [6], though incidence was much higher in China, India and United States [3, 6]. Female youth are disproportionately affected, possibly reflecting pubertal sex differences in adiposity, physical activity, sleep, mental health and insulin resistance [2, 7–11], as well as differential responses to in-utero hyperglycemia between female and male offspring [12]. Delayed diagnoses may also disproportionately affect young males due to lower levels of engagement with health services than females [1, 13].

Internationally, pre-diabetes prevalence is also high [14]. United States (US) data from 2005 to 2016 suggested that nearly 20% of adolescents had pre-diabetes [15], with higher rates among those with obesity. Screening of asymptomatic children and adolescents in New Zealand [16] and India [17] identified similar rates, again noting risk factors of obesity, central adiposity, sedentary lifestyle and ethnicity. However, elevated fasting glucose was predominant in some studies [14], possibly reflecting non-fasting status versus true pre-diabetes.

**Fig. 1:**
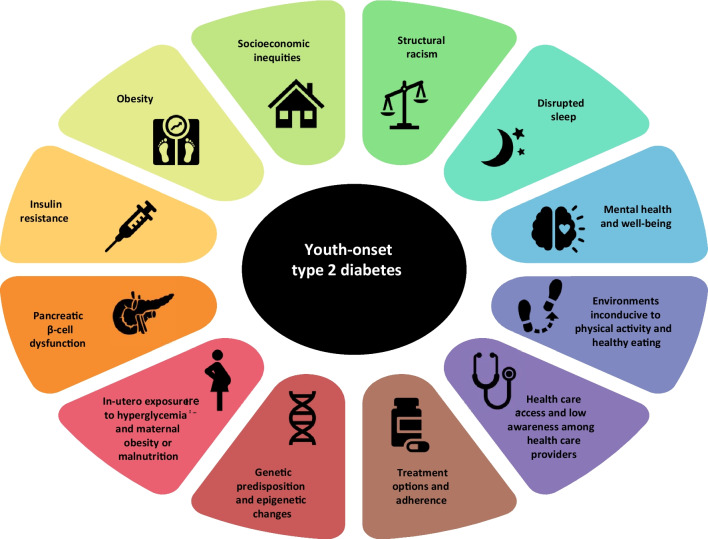
Factors contributing to risk and severity of youth-onset T2D

In addition to increasing prevalence, the future trajectory of YO-T2D is of high concern. The pathophysiology, phenotype, comorbidities, complications and treatment response all appear significantly worse than later-onset diabetes, raising serious concerns for the future health of affected youth [1]. In particular, glycemia is chronically suboptimal in YO-T2D, with only 14% meeting glycemic targets [18], 34% having HbA1c > 10% (86mmol/mol) at 15 years post-diagnosis [19], and a high prevalence of complications occurring within this period [19]. Thus, YO-T2D has been described as a “severe aggressive phenotype” [1] (Fig. 1), posing a public health challenge regarding optimal prevention, screening and treatment to address the significant long-term health impacts [2, 20].

Of concern, YO-T2D particularly affects marginalized and socioeconomically vulnerable youth [3, 21], considered an avoidable “disease of poverty” [21], now exceeding type 1 diabetes (T1D) diagnoses in some populations. High-risk ethnicities include First Nations, African-American, Hispanic, Pacific Islander, Asian and Middle Eastern populations [3, 4, 6, 22–24]. YO-T2D reflects underlying structural and social inequities [21, 25]. These inequities impact both the risk of developing YO-T2D, as well as access to culturally-safe health care, food security, risk of diabetes-related complications, and clinical management [25]. Consequent to these inequities, T2D medications are understudied in youth. Worldwide, only approximately 2% of youth with T2D are eligible and able to participate in a randomised controlled trial [20], despite affected youth being at high risk secondary to chronic hyperglycemia and a high prevalence of complications [19].

This review will explore the emerging evidence regarding pathogenesis, future trajectories, treatment options, and phenotypes of YO-T2D. There is an urgent need to define which youth are at higher risk of developing YO-T2D, develop effective interventions for prevention, and address intergenerational transmission in high-risk populations.

## Defining the Issues

There are limitations in using simple measures to define insulin resistance in youth due to the lack of universally agreed normal ranges for insulin levels, considering the physiological insulin resistance of puberty [26, 27]. Therefore, assessing clinical evidence of insulin resistance such as non-alcoholic fatty liver disease (NAFLD), low HDL-cholesterol, and high triglycerides is important, because increased adiposity and insulin resistance have an additive effect on cardiovascular risk [28].

The term ‘pre-diabetes’ is not yet universally accepted in youth despite its wide use in adult guidelines [29, 30]. Adult criteria for pre-diabetes include fasting plasma glucose 100-125mg/dL (5.6–6.9 mmol/L), also called impaired fasting glucose (IFG), HbA1c 5.7–6.4% (38-46mmol/mol), or 2-h post-load plasma glucose 140-199mg/dL (7.8- 11.0mmol/L), also called impaired glucose tolerance (IGT) [31]. However, there are concerns that extrapolating adult data to youth is inaccurate in predicting risk of future T2D or diabetes complications [32, 33]. In the US HEALTHY study, 2% of normal weight youth, without any T2D risk factors, had HbA1c ≥ 5.7% (38mmol/L) with overlap in HbA1c distribution between youth with normal weight versus overweight/obesity [34]. Potentially lower HbA1c cut-offs should be used to define pre-diabetes in youth with overweight/obesity in terms of correlation with IFG or IGT [35].

Diagnostic criteria for diabetes in youth include fasting plasma glucose ≥ 126mg/dL (7.0 mmol/L), glycated haemoglobin (HbA1c) ≥ 6.5% (48mmol/mol), or 2-h post-load plasma glucose ≥ 200mg/dL (11.1 mmol/L) [29].

## Pathogenesis

Our understanding of the underlying mechanisms leading to YO-T2D continues to evolve (Fig. 1). YO-T2D is secondary to both insulin resistance, in the liver, adipose tissue and peripheral tissues, and now increasingly recognized secondary to pancreatic β-cell dysfunction leading to reduced insulin production over time, and consequent relative insulin deficiency [36]. Youth with T2D also have more rapid deterioration of β-cell function post-diagnosis [37] than those with later-onset T2D [38]. Insulin resistance, while clearly present at diagnosis, appears to remain relatively stable over time post-diagnosis, while β-cell function continues to decline, in contrast to later-onset T2D [37, 39, 40]. More severely deteriorating β-cell function may underly the rapid loss of glycemic control, higher HbA1c and ‘more severe aggressive phenotype’ of YO-T2D [1]. Co-existent insulin resistance from obesity and physical inactivity, may accelerate or worsen this situation by increasing insulin secretion, and potentiating β-cell failure.The Restoring Insulin SEcretion (RISE) study has been important in increasing our understanding of these mechanisms by allowing for direct comparisons between youth and adults with pre-diabetes and recently diagnosed T2D [43]. Β-cell decline continued in youth regardless of treatment, and insulin sensitivity was lower for similar body mass index (BMI) [41], but youth required increased insulin secretion in response to a glucose load compared to adults [44].

Pubertal insulin resistance is likely also contributory [41], and may explain why T2D is still relatively uncommon pre-puberty, and why onset of YO-T2D is often younger in girls, whose puberty onset is younger than boys[42]. There is evidence that intergenerational transmission of T2D leads to earlier pre-pubertal diagnosis in the offspring of mothers with T2D, with children who are diagnosed pre-pubertally tending to have in-utero exposure, greater metabolic abnormalities and/or come from higher-risk populations [42, 45]. While YO-T2D is a heterogenous disease, there is likely a difference in risk factors, pathogenesis and complications between youth diagnosed before and after puberty [45, 46]. Puberty may therefore be important in considering timing of screening, and identifying risk (see below). Stage of puberty at diagnosis may also impact trajectory of disease [46] and influence treatment options [20] (see below).

**Fig. 2:**
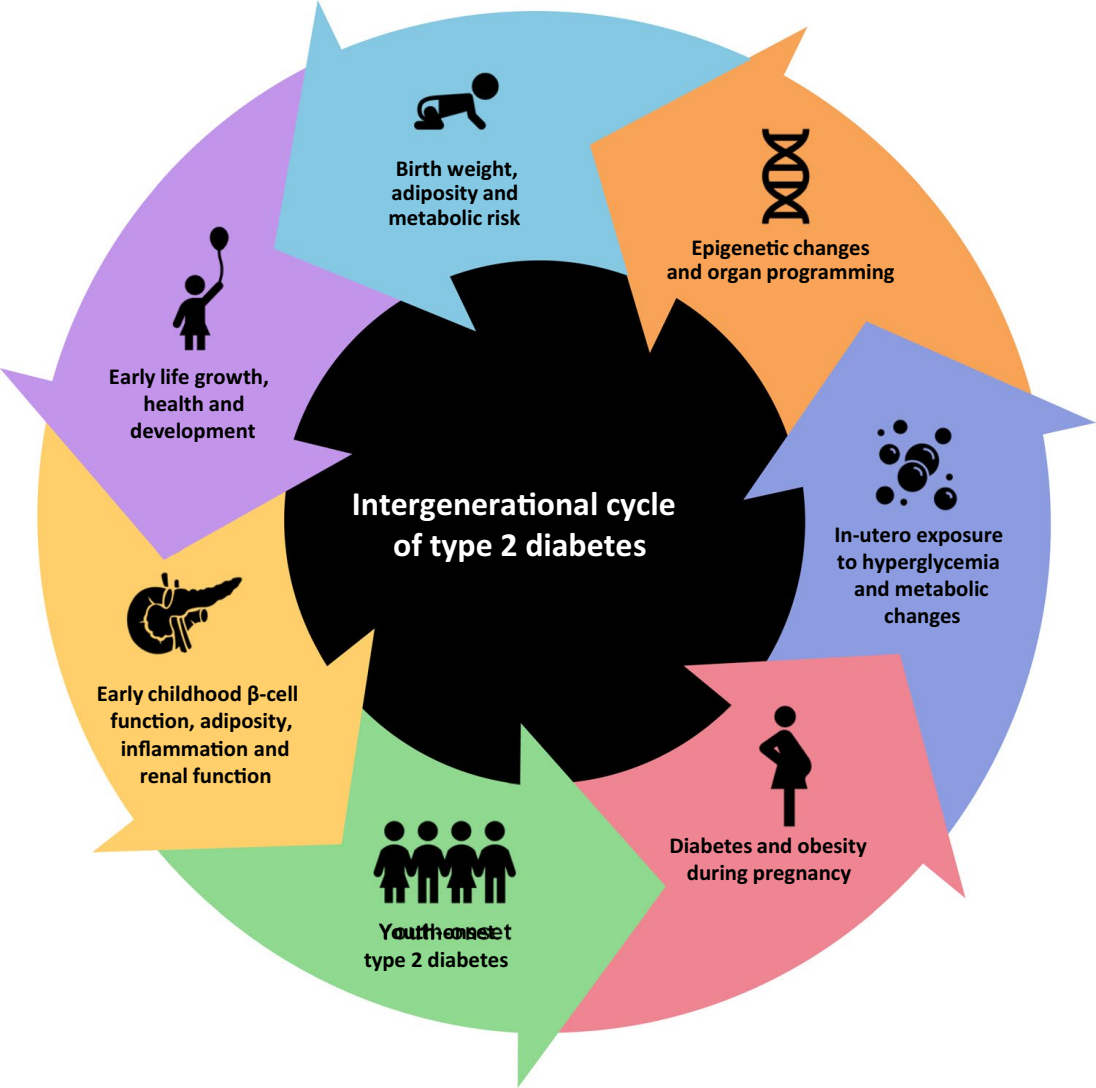
Intergenerational transmission of risk for youth-onset T2D

A low Disposition Index (DI, i.e. insulin secretion relative to insulin sensitivity) is an early marker of impaired β-cell function and, while reversible, may predict future development of diabetes [47]. US data suggest that DI is an important predictor of T2D in youth with obesity, and that the most significant predictor tested to-date is exposure to in-utero hyperglycemia [48]. This relationship varies with maternal race/ethnicity, independent of maternal BMI [49]. The in-utero environment, particularly exposure to maternal obesity, nutrient imbalance, and hyperglycemia, is important in risk of diabetes and obesity among offspring, leading to intergenerational transmission in high-risk populations [50–52] (see below and Fig. 2), though the underlying mechanisms are still incompletely understood. Maternal malnutrition in pregnancy and nutritional deficiencies in early childhood likely increase risk through multiple interconnected pathways, including changes in the gut microbiome, chronic inflammation, altered insulin signaling and metabolic parameters [53].There is evolving evidence regarding the role of micronutrients in YO-T2D pathogenesis. Vitamin D and A sufficiency may be potentially protective in reducing inflammation and maintaining insulin secretion and β-cell mass, and magnesium and selenium may act as antioxidants, improving β-cell function and/or insulin sensitivity [54]. This suggest that both macro- and micro- nutrient sufficiency are important in reducing risk of YO-T2D.

Consistent with the concept of insulin resistance, the increase in YO-T2D is mirrored by an increase in obesity in youth worldwide [55]. Potential contributory factors include obesogenic social and food environments, including food advertising, pricing and availability, reduced opportunity for physical activity and increasingly sedentary lifestyles, increased use of cars for transport, concerns regarding community safety and walkability, inadequate sleep, and mental health issues such as depression and disordered eating [56–58]. Worldwide, obesity is more common in YO-T2D than later-onset T2D [41, 58], those with YO-T2D are less likely to be of White race than later-onset T2D [1, 4, 41], and BMI is inversely related to age at T2D diagnosis [59]. Central and visceral adiposity is also a predominant feature of YO-T2D [60].

There are varying phenotypes of YO-T2D, however, and not all affected youth have overweight or obesity [61], particularly in high-risk populations with a high background prevalence of T2D, such as Canadian First Nations youth with G319S mutation of the HNF-1α gene [42]. In-utero exposures likely play a role, leading to epigenetic changes and in-utero organ programming. This may cause loss of β-cell function, consequently increasing risk of YO-T2D and a shifting phenotype to a lower BMI at presentation from what has been seen historically [61, 62]. In these contexts, waist-to-height ratio, as a marker of central adiposity, may be of more use clinically than BMI [63, 64]. These differing phenotypes are still poorly understood, and require more research to better target medications to preserve or improve β-cell function in affected youth. For example, the G319S mutation causes defective insulin secretion, producing YO-T2D at a lower BMI than First Nations Canadian youth with T2D without the mutation [65]. Since this landmark finding more than 20 years ago, more than 65 other genetic variants have been identified which increase T2D risk, including 7 in YO-T2D [66]. However, each of these newer variants are thought to only have a small impact on risk and are not yet part of routine clinical practice.

## Intergenerational transmission of risk (Fig. 2)

Studies among the Pima peoples first demonstrated the impact of hyperglycemia in pregnancy [67], with obesity risk in offspring increasing from 5 years of age. By 25–34 years of age, the prevalence of T2D was 70% among offspring of mothers with T2D in pregnancy, compared to < 15% in offspring of mothers without hyperglycemia [67]. Offspring of mothers with T2D were almost three times as likely to have obesity in childhood than offspring of mothers without hyperglycemia [68]. The effect of in-utero exposure to maternal diabetes on the risk of T2D in offspring appeared additive to any underlying genetic susceptibility [61].

The US Search for Diabetes in Youth (SEARCH) study suggested a striking 47% of T2D in youth could be attributed to intrauterine exposure to maternal diabetes and obesity [51]. However, there was only 4.7% attributable risk in youth exposed only to hyperglycemia in-utero without obesity, and 19.7% attributable risk in youth exposed to maternal obesity in-utero without hyperglycemia. This suggests an additive effect and that prevention of maternal hyperglycemia, obesity and other potential factors coexisting with T2D/gestational diabetes (GDM) are all important targets for intervention. A Canadian cohort demonstrated that maternal T2D has greater risk for offspring than GDM, possibly because hyperglycemia exposure occurs during fetal organogenesis[50]. However, there is also an increased risk of IGT [69–71], insulin resistance [48], pancreatic β-cell dysfunction [48, 49, 72], obesity [73] and metabolic syndrome [73] in offspring of women with GDM compared to normoglycemic pregnancies.

Of note, much of the available evidence regarding intergenerational transmission of YO-T2D comes from high-risk populations, with little known regarding timing of the critical period for exposure to hyperglycemia, impact of other co-existing intrauterine factors relating to insulin resistance such as hyperinsulinemia and hypertriglyceridemia/elevated FFAs, or impact of T2D treatment on future risk [74]. It is also difficult to measure general societal changes contributing to increased prevalence of cardiometabolic conditions in some populations [75]. Multiple studies, however, have demonstrated much higher risk for offspring of mothers with T2D compared to fathers [49, 68, 71]. The ‘thin-fat’ phenotype described in some populations, involving preferential growth of adipose tissue compared to lean mass in children exposed to hyperglycemia in-utero[76–78], compounded by maternal malnutrition and nutrient imbalance [52], is also likely influential [61, 62]. Children exposed to the double burden of both nutritional deficiency and dietary excess at various time points during pregnancy and their lifecourse, have increased cardiometabolic risk [53].

Epigenetic changes are also likely important in understanding increasing diabetes rates in high-risk populations [79, 80]. Changes in DNA methylation may be induced by the in-utero environment, with lifelong metabolic risk possibly influenced by the timing of exposure, although limited longitudinal data are currently available [81, 82]. Moreover, among youth in the Treatment Options for Diabetes in Adolescents and Youth (TODAY) study who became pregnant, 21% had major congenital anomalies (4 times the rate reported in adult-onset T2D), of high concern for health of offspring of mothers with YO-T2D[83].

## Future Trajectories

**Fig. 3:**
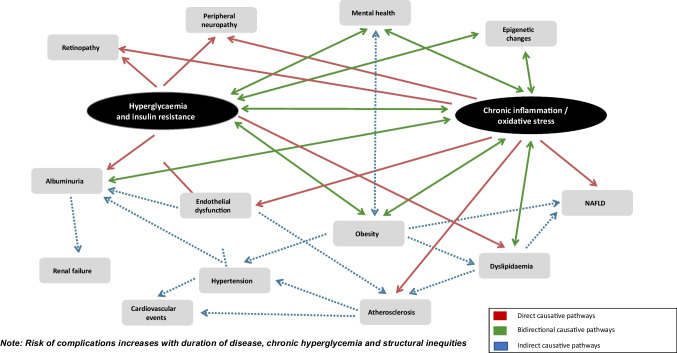
Pathogenesis of comorbidities and long-term complications of youth-onset T2D—causative pathways from hyperglycemia and oxidative stress to cardiometabolic complications

YO-T2D is associated with increased rates of complications compared to later-onset T2D [1, 19, 84], even after controlling for duration of diabetes (Fig. 3). However, longer periods of exposure to hyperglycemia also compound the impact of diabetes complications [20] and YO-T2D is associated with more severe hyperglycemia than adult-onset T2D [19]. Complications are also more likely to be present at the time of diagnosis in YO-T2D [1], and recent TODAY follow-up data demonstrated that the majority of youth will have complications by 15 years post-diagnosis [19]. At baseline, 8% of youth in TODAY had diabetic kidney disease (DKD), increasing to 54.8% by 15 years, 19.2% had hypertension (increasing to 67.5%) and 20.8% had dyslipidemia (increasing to 51.6%). At 15 years post-diagnosis, 80.1% had at least one microvascular complication, including 35% with peripheral neuropathy, and 50% with retinopathy. The SEARCH study also demonstrated that complication risk is much higher in YO-T2D than T1D [85], even after adjustment for socioeconomic factors. This finding has been replicated in other populations [2, 84].

DKD, defined by albuminuria (> 30µg/mg) or estimated glomerular filtration rate < 60ml/min/1.73m^2^ is far more common in YO-T2D than T1D [85, 86]. Canadian data demonstrated that 45% of patients with YO-T2D who continued follow-up to 20 years post-diagnosis required renal replacement therapy, compared to none of those with T1D [86]. YO-T2D was also associated with a 23 times higher risk of renal failure and 39 times higher risk of need for dialysis, compared with youth without diabetes [86]. Albuminuria occurs earlier post-diagnosis in YO-T2D than adult-onset T2D [87], sometimes being present even at diagnosis, and BMI appears important in the progression of renal disease in YO-T2D [88]. The risk of progression to renal failure varies between populations, however, possibly relating to underlying population risk of renal disease or in-utero programming [2, 87].

Multiple studies have demonstrated early morbidity and mortality with YO-T2D [2, 89–91]. In Australia, a 10 year earlier diagnosis of T2D was associated with 20–30% increased risk of all-cause mortality and 60% increased risk of cardiovascular mortality [90]. Another study suggested three times higher mortality for those diagnosed with T2D between 15 and 20 years of age versus the general population [92]. Cardiovascular disease appears to be the major contributor to increased mortality even at a young age [19], including 17 serious cardiovascular events by 15 years-post diagnosis (average age 29 years) in the TODAY study [19], and at a far greater rate than in T1D [84, 89].

## Screening for T2D and Pre-diabetes

Although screening for T2D in adults with risk factors helps prevent progression to T2D and its complications [93], there is limited evidence among youth [29, 30]. It is well established that some proportion of youth with pre-diabetes will have normalisation of glycemia without intervention and no study has thus far directly assessed the benefits or harms of screening [94]. In low-risk populations, screening youth with overweight or obesity for T2D is unlikely to be cost-effective. As evidence, in the US, screening youth with obesity and ≥ 1 other risk factor for T2D identified < 0.5% with previously undiagnosed T2D [95]. There is also limited evidence as to the value of screening in high-risk populations [29]. School based urinary glucose screening in Japan between 1975–2015 had a diagnostic pick up rate of only 0.01–0.02% [96]. The 2022 International Society for Pediatric and Adolescent Diabetes (ISPAD) guidelines suggest a risk-factor based strategy for screening from 10 years of age, using BMI ≥ 85th percentile for age in addition to ≥ 1 other risk factor, with screening repeated 3-yearly if risk continues [29]. These guidelines have not specified a recommended test between fasting glucose, oral glucose tolerance test, or HbA1c. Canadian guidelines stratify determination of need for screening by pubertal status, recommending that pre-pubertal children be screened if they have ≥ 3 risk factors, compared to ≥ 2 risk factors in pubertal youth [97].

Other than in very high-risk populations with a high background prevalence of T2D and known intergenerational transmission of cardiometabolic conditions, such as First Nations communities, T2D remains rare pre-puberty [98, 99]. However, populations affected are changing over time, and 25.3% of those with youth-onset diabetes in the Indian registry now having T2D [100]. In high-risk populations, annual screening using HbA1c of youth with any risk factor is likely to be valuable, as currently recommended in Australia and New Zealand [101]. BMI may poorly identify high-risk youth in these populations [61, 78], and use of waist-to-height ratio should be considered [101].

Screening for pre-diabetes and insulin resistance in youth is also controversial, acknowledging that the current diagnostic criteria for pre-diabetes have not been specifically validated in youth [29, 30], and that physiological insulin resistance occurs in puberty. In following youth with IGT at baseline for up to 10 years, the reported incidence of T2D has ranged between 1 to 56% [102]. In a large US study, HbA1c had a stronger and more specific association with cardiometabolic risk in youth than IFG [95], particularly in high-risk ethnicities [34]. Moreover, many youth with overweight/obesity and prediabetes revert to normoglycemia during follow-up, especially in lower ranges of glucose elevation (HbA1c < 6.0%) [103]. Data on the accuracy of various risk assessment tools developed to help estimate the risk of T2D among youth with pre-diabetes remain inadequate [30]. Unresolved questions remain regarding timing and method of screening, as well as the design of effective interventions or treatments to prevent progression to T2D [104]. While oral glucose tolerance testing is likely not feasible for screening, it is more likely to detect youth with early stages of glucose dysregulation compared to HbA1c [29], should effective interventions eventually arise.

## Clinical Management

After a diagnosis of T2D, individualised self-management education that is age and developmentally appropriate, culturally-relevant, and family-centred should be provided, aiming for supported behavioural change, and preferably delivered by a multidisciplinary team [29]. There is growing awareness of the impact of stigma and shame regarding a diagnosis of T2D on subsequent self-management [105, 106], as well as the socioeconomic inequities faced by many youth with T2D [21]. Collaborations with schools and community providers are therefore important in improving outcomes [29, 101], as well as consideration of alternative clinical approaches and behavioural therapies [107]. A combination of lifestyle modification and medications is recommended, particularly noting the importance of regular physical activity in improving glycemia and reducing cardiovascular risk [29, 108]. Exercise improves multiple parameters of glycolipid metabolism, including uptake and utilisation of glucose and lipids, modulation of DNA methylation, slowed decline in pancreatic β-cell function, reduced oxidative stress, and improved insulin sensitivity [109].

There is also increasing awareness of the need to consider mental health and sleep in terms of risk of developing T2D [11], especially in girls who report higher rates of depression and disordered eating and fewer hours of sleep than boys [9, 10]. These factors also affect quality of life [110], capacity for self-management [107], and risk of developing complications, particularly renal [111]. The association between renal complications and mental health appears to be mediated by increased inflammation [111]. Canadian data comparing children with T2D to matched peers indicated that those with T2D were 2.38 times more likely to have a mood or anxiety disorder before diagnosis and 3.18 times more likely to attempt/complete suicide [112]. Hong Kong data demonstrated 36.8% of admission bed-days in those with YO-T2D to be due to mental illness[110]. Improved sleep quality and increased sleep time may improve insulin sensitivity in adolescents [113], as well as impacting on eating behaviours, physical activity and sedentary time [114]. It is also important to consider risk behaviours, such as smoking and alcohol use, that may affect wellbeing and further increase diabetes-related cardiovascular risk [29].

International guidelines vary regarding glycemic targets in YO-T2D, with ISPAD [29] and American Diabetes Association [115] guidelines suggesting target HbA1c of < 7% (53mmol/mol) and the Australian and New Zealand Society for Paediatric Endocrinology and Diabetes [101], National Institute for Health and Care Excellence [116], and 2024 United Kingdom consensus[117] guidelines suggesting < 6.5% (48mmol/mol). Youth with T2D respond differently to pharmacological management than later-onset T2D, with a higher rate of treatment failure compared to adult cohorts to-date [1, 37]. This likely relates to the rapid β-cell dysfunction associated with YO-T2D. Mental health and socioeconomic factors also contribute to lower engagement and adherence with treatments [105, 118, 119], exacerbated by sub-optimal knowledge amongst health care providers regarding YO-T2D [36, 120, 121] and lack of access to informed, culturally-appropriate care settings [21, 105, 106].

The TODAY study in the US was the landmark study in this area, with a median time of only 11.5 months to treatment ‘failure’, and 51.7% of youth experiencing loss of glycemic control on metformin alone [122] vs. 12% of adults in the ADOPT study with the same duration of metformin treatment [123]. HbA1c ≥ 6.3% at 2 months post-diagnosis was predictive of treatment failure, suggesting early consideration of addition of other agents in recent international guidelines [20, 116, 117]. Multiple studies have demonstrated minimal effectiveness of lifestyle modification interventions in improving health behavior in YO-T2D [107, 124]. While YO-T2D disproportionately affects females, the lifestyle intervention in the TODAY study concerningly had reduced effectiveness and adherence in females [122]. The RISE study demonstrated that the main predictor of worsening glycemia in adolescents with T2D or IGT was baseline β-cell dysfunction, and that neither metformin nor insulin plus metformin prevented deterioration in β-cell function in those with T2D or pre-diabetes, in contrast to adults [38, 44].

In recent years, there has therefore been growing awareness of the need to expand medication options for youth with T2D, despite the paucity of data [20]. Further work is required to determine optimal pharmacotherapy for varying phenotypes of YO-T2D, and to determine effective methods of maintaining or improving β-cell function. Metformin remains the first-line treatment [29], acting primarily on liver and skeletal muscle to stimulate glucose uptake and reduce glucose production. However, gastrointestinal side effects often limit dosing. Insulin is recommended as first line when initial HbA1c ≥ 8.5% (69mmol/mol) [29], though there is increasing consensus regarding the need for other medications long-term [20, 116, 117], especially given the weight gain seen in youth treated with insulin in RISE [125].

Weekly glucagon-like peptide-1 receptor agonists (GLP-1-RA) are often prescribed off-label for youth though there is growing evidence of their efficacy and safety in YO-T2D. They are also now recommended in some guidelines [116, 117] as the second line therapy of choice if metformin monotherapy fails to achieve glycemic targets, in preference to long-term insulin. The first such study explored daily liraglutide, demonstrating a 1.3% reduction in HbA1c at one year, and 63.7% achieving HbA1c < 7% (53mmol/mol), compared to only 36.5% of the metformin/insulin placebo group [126]. In the AWARD-PEDS study, HbA1c was 1.4% lower at 26 weeks in the weekly dulaglutide group versus metformin/insulin placebo [127]. A smaller study examining weekly exenatide suggested 0.85% reduction in HbA1c compared to placebo (metformin or sulphonylurea and/or insulin) [128]. Real-world analyses confirm efficacy of GLP-1-RA in youth [129], though interestingly all studies to date have demonstrated minimal impact of GLP-1-RA on BMI of youth with T2D, versus adults [126–128, 130]. Possible explanations include more severe hyperglycemia seen in youth with T2D at diagnosis versus adults, faster glycemic failure and increased insulin resistance, or lower medication adherence [127]. Of note, the GLP-1-RA semaglutide was effective in weight loss for adolescents with obesity without T2D [131], similarly to adults, with 16.1% weight loss at 68 weeks in the semaglutide group compared to 0.6% in placebo group. Maximal follow-up thus far in GLP-1-RA studies in youth is only 68 weeks, so it remains unclear whether there are similar cardiovascular, hepatic and renoprotective benefits as in adults [36]. The STEP-TEENS study of youth with obesity without T2D demonstrated improvement in dyslipidaemia and hepatic steatosis by 68 weeks [131].

Sodium-glucose cotransporter 2 inhibitors (SGLT-2i) are also prescribed off-label in youth. There are conflicting data regarding impact on glycemia, though they appear similarly renoprotective as in adults, attenuating diabetes-induced transcriptional changes in metabolic pathway genes [132] and reducing intraglomerular pressure [133]. One study demonstrated no effect of 10mg dapagliflozin on HbA1c versus placebo at 24 weeks in intention to treat analysis, though protocol-adherent patients had a 1.13% reduction [134]. This is consistent with two further studies demonstrating 1.03% reduction in HbA1c with 5mg dapagliflozin versus placebo at 26 weeks [135], and 0.84% lower HbA1c using empagliflozin versus metformin/insulin at 26 weeks [136]. No study has demonstrated changes in weight or blood pressure with SGLT-2i in YO-T2D and the long-term safety profile is unknown.

Other classes of diabetes medications are not recommended for use in YO-T2D. Dipeptidyl peptidase IV inhibitors have showed no impact on HbA1c versus metformin/insulin placebo in multiple randomised controlled trials [135–138]. Sulphonylureas are not recommended because of theoretical potentiation of β-cell decline in YO-T2D [36], and increased weight gain and hypoglycemia versus metformin [139]. While rosiglitazone, a thiazolidinedione, was shown to reduce risk of glycaemic failure by 13% compared to metformin in the TODAY study [122], it is not recommended for use in YO-T2D due to increased fracture and heart failure risk in adults [140] and a smaller increase in bone mineral density and content in TODAY’s metformin + rosiglitazone group versus metformin or metformin + intensive lifestyle at 24 months [141], though echocardiogram findings did not differ by treatment group [142]. However, the thiazolidinedione pioglitazone is increasingly used off-label in YO-T2D [20] in light of possible improvement in insulin sensitivity and NAFLD potentially outweighing theoretical risks.

There is also increasing interest in the role of metabolic bariatric surgery (MBS) in YO-T2D. The Teen-LABS study suggested that MBS is more effective in youth than adults regarding remission of T2D (95% in youth vs. 53% in adults), and improving obstructive sleep apnoea, NAFLD, dyslipidemia (66% remission), DKD (86% remission) and hypertension (74% remission) [143]. Participating youth had a mean weight loss of 27% at 3 years. A retrospective comparison of Teen-LABS and TODAY study data, acknowledging differences in participant characteristics and study design, suggested that MBS may offer benefits over medical treatment in some phenotypes of YO-T2D, including risk of diabetes-related complications [144, 145]. Thus far, there are minimal differences in MBS risks or outcomes between older and younger adolescents [146], suggesting that determining appropriateness for MBS should not be solely determined by age. However, few youth in Teen-LABS had T2D [143], and nearly all underwent Roux-en-Y gastric bypass versus the now dominant vertical sleeve gastrectomy (VSG) procedure, necessitating further study of VSG in YO-T2D.

Another important consideration in management of YO-T2D is pubertal stage. While there is very limited evidence thus far regarding the safety profile and benefit of newer medication options in pre-pubertal youth [20], decisions regarding the use of off-label medications need to also consider the long-term effects of chronic hyperglycemia. As discussed previously, insulin resistance inherent to puberty contributes to development of YO-T2D. However, pubertal progression may also impact on the trajectory of YO-T2D. Pubertal insulin resistance, growth factors and hormonal changes may interact to accelerate progression of renovascular disease [46, 147]. Mechanisms remain unclear though are likely additive, such as i) rapid kidney growth in puberty increasing susceptibility to oxidative stress; ii) effects of pubertal increases in growth hormone and insulin growth factor-1 on renal tubular filtration; iii) effect of pubertal insulin resistance on sodium reabsorption and energy production pathways within the kidney. The higher risk of DKD in YO-T2D than later-onset T2D is also likely a consequence of the more severe dysglycemia and β-cell loss in YO-T2D, leading to further glomerular hyperfiltration, dysregulated energy pathways within the kidney, and oxidative stress [46]. Albuminuria and hypertension therefore need to be closely monitored, with a low threshold for treatment.

## Conclusion

YO-T2D is increasing in prevalence worldwide and has a more severe and aggressive phenotype than adult-onset T2D, associated with poorer response to the limited currently available treatments and a high risk of diabetes-related complications at a young age. The evidence suggests that the pathophysiology of YO-T2D differs from adult-onset T2D, highlighting the urgent need to better understand who is at risk for YO-T2D, the differing phenotypes, and to effectively target prevention and treatments. YO-T2D disproportionately affects socioeconomically disadvantaged youth and intergenerational transmission among high-risk communities will lead to worsening health outcomes and perpetuation of inequity if left unchecked. There is also an urgent need to develop new models of care that more effectively engage youth with T2D and holistically improve mental health and wellbeing.
